# Unchanged Cognitive Performance and Concurrent Prefrontal Blood Oxygenation After Accelerated Intermittent Theta-Burst Stimulation in Depression: A Sham-Controlled Study

**DOI:** 10.3389/fpsyt.2021.659571

**Published:** 2021-06-30

**Authors:** Wiebke Struckmann, Jonas Persson, Malin Gingnell, Wojciech Weigl, Caroline Wass, Robert Bodén

**Affiliations:** ^1^Psychiatry, Department of Neuroscience, Uppsala University, Uppsala, Sweden; ^2^Department of Psychology, Uppsala University, Uppsala, Sweden; ^3^Anaesthesiology and Intensive Care, Department of Surgical Science, Uppsala University, Uppsala University Hospital, Uppsala, Sweden; ^4^Department of Pharmacology, University of Gothenburg, Göteborg, Sweden

**Keywords:** cognition, fNIRS, iTBS, repetitive transcranial magnetic stimulation, rTMS

## Abstract

**Aim:** Intermittent theta-burst stimulation (iTBS) delivered over the dorsomedial prefrontal cortex (DMPFC) has shown promise as a treatment for anhedonia and amotivation in patients with depression. Here, we investigated whether this protocol modulates cognitive performance and concurrent prefrontal blood oxygenation. We also examined whether depressed patients exhibit cognitive dysfunction and prefrontal hypoactivity at baseline compared to healthy controls.

**Methods:** This sham-controlled study comprises 52 patients randomized to either active or sham accelerated iTBS over the DMPFC (applied twice daily) for 10 consecutive treatment days, and 55 healthy controls. Cognitive performance was assessed at baseline and once again 4 weeks later using a cognitive test battery targeting attention, inhibitory control, and numerical, verbal, and visual working memory. Concurrent prefrontal oxygenated hemoglobin (oxy-Hb) was captured with functional near-infrared spectroscopy.

**Results:** Active iTBS over DMPFC did not affect cognitive performance or concurrent oxy-Hb change compared to sham iTBS in patients with depression. Compared to controls, patients at baseline showed impaired performance in the Trail Making Test, the Rey Auditory Verbal Learning Test, the Animal Naming Test, and the Digit Symbol Substitution Test, however no difference in prefrontal oxy-Hb was observed.

**Conclusion:** Patients with treatment-resistant depression displayed cognitive deficits, however without prefrontal hypoactivity, compared to healthy controls at baseline. iTBS treatment did not alter cognitive performance, nor concurrent prefrontal blood oxygenation, in patients. Taken together, iTBS can likely be considered a cognitively safe treatment option in this sample of patients.

## Introduction

Depression, the mental health condition with the highest global disease burden (1), is characterized by persistent low mood, loss of interest, and low energy (2). Depression is also accompanied by impaired cognitive functions, in domains such as processing speed, working memory, and episodic memory (3). It is crucial to understand the neurobiological underpinnings of cognitive symptoms in depression, such as prefrontal hypoactivity (4), to develop appropriate treatment options.

For treatment-resistant depression, there are some treatment alternatives, with electroconvulsive therapy (ECT) being the most effective. ECT is a neuromodulatory therapy with global impact on the brain with high remission rate but also cognitive side effects (5). Repetitive transcranial magnetic stimulation (rTMS) over the dorsolateral prefrontal cortex (DLPFC) is an established add-on treatment alternative, with a more focal brain impact (6). The DLPFC is a node in the central executive network (7) and given that rTMS modulates local excitability over the DLPFC this in turn has effect on network signaling (8). There were early concerns that rTMS over the DLPFC might have cognitive side effects as well, however this could not be confirmed (9). While rTMS has been shown to potentially even cause cognitive enhancement in healthy subjects (10), a recent meta-analysis on depression and DLPFC-rTMS protocols found only modest task-specific cognitive improvement after treatment, and this only for the Trail Making Test (11). Using the shorter intermittent theta-burst stimulation (iTBS) (12) over the DLPFC in treatment-resistant depression, a sham-controlled study suggests cognitive improvement also in the Wisconsin Card Sorting Test (13).

While rTMS is effective in treatment-resistant depression, some of the symptoms, such as anhedonia and amotivation, have been less successful to treat with conventional rTMS protocols targeting the DLPFC (14). Anhedonia and amotivation are symptoms that cross over diagnostic boundaries and might have a common origin from an emotional and cognitive network that may be reached by instead targeting the dorsomedial prefrontal cortex (DMPFC) (15). Based on its suggested involvement in a depression network, the DMPFC has been proposed as an alternative rTMS target (16). An initial open-label study has indeed shown that the DMPFC is well-suited for rTMS treatment of depression (17). This target might be of interest also in the context of cognition, as DMPFC and DLPFC are involved in the same cognitive control network, with the DMPFC monitoring cognitive performance and the DLPFC adjusting behavior (18).

In a recent randomized clinical trial with a transdiagnostic approach, accelerated iTBS delivered twice daily over the DMPFC in patients with uni- or bipolar depression or schizophrenia conducted by our group (19), we found a treatment effect only in patients with depression and specifically for negative symptoms such as anhedonia and amotivation, but not for depressive symptoms overall. While we found that active and sham iTBS were similar in their reports of subjective memory deficits, fatigue, and headache, we do not yet know about the effects of this specific protocol on actual cognitive performance. The cognitive effects of iTBS over DMPFC in depression have been assessed in an open-label case series which suggests cognitive safety with task-specific improvement for the Trail Making Test and the Stroop Test (20), but no sham-controlled studies have been published yet.

Functional near-infrared spectroscopy (fNIRS) is a non-invasive technique that can be used to measure changes in cortical oxygenated hemoglobin levels (oxy-Hb) (21). Due to its relative insensitivity to body motion, fNIRS is well-suited to be applied during cognitive tests. This technique has indeed been used to capture changes in oxy-Hb during cognitive performance in patients with depression, such as prefrontal hypoactivity (22, 23). A sham-controlled trial of iTBS for panic disorder investigated changes in the prefrontal fNIRS signal during a verbal fluency task (24) and the Emotional Stroop Test (25), but to our knowledge there are yet no studies of depression in this regard.

In the present study, we explored whether accelerated iTBS delivered over the DMPFC in depression affected cognitive performance in a neurocognitive test battery, as well as the concurrent blood oxygenation response by applying prefrontal fNIRS during cognitive testing. The fNIRS signal was recorded from sites roughly corresponding to the left and right DLPFC, thus probing for indirect effects on the cognitive control network. We also examined whether patients at baseline exhibited cognitive dysfunction and prefrontal hypoactivity as measured with oxy-Hb response, during cognitive testing, compared to a group of healthy controls.

## Materials and Methods

### Participants

This study comprised 52 patients from two randomized controlled trials with shared methodology at the Brain Stimulation Unit, Uppsala University Hospital, Sweden. Parts of this data has been reported elsewhere (19). The current patient sample included all 40 patients from our previous study (19) plus additional 12 patients with depression from an add-on study. Patients were recruited via the psychiatric clinic at Uppsala University Hospital, Sweden and were required to have a current depressive episode in either a uni- or bipolar disorder and unchanged psychotropic medication 1 month before treatment start. Patients' current medication was kept constant throughout the study and the study protocol did not allow benzodiazepine use. Data from the patient group were also compared with a cohort of 55 healthy controls recruited via advertisement over the internet. An overall inclusion criterion was age 18–59. Exclusion criterion for the control group was any ongoing, or history of, psychiatric disorder, and for all participants epilepsy, intracranial metallic implants, pacemaker or implantable cardioverter defibrillator, pregnancy, or an active substance use disorder. All participants underwent a Mini International Neuropsychiatric Interview (M.I.N.I.) (26). Comorbid clinical diagnoses of attention deficit hyperactivity disorder (ADHD) or attention deficit disorder (ADD) were collected from the medical records. Written informed consent was obtained from all participants. The study was approved by the Ethical Review Board, Uppsala University and performed in accordance with the principles of The Declaration of Helsinki.

### Procedures

Patients were randomized to either active or sham iTBS treatment. The study was double-blinded, with patients and the symptom assessors being blinded to the treatment allocation. To ensure blinding, the magnetic stimulator operating nurse received a randomization code for each patient, prepared by an independent research organization. The code was entered in the stimulator research software upon each treatment session, which then directed the operator which side of the TMS coil to be angled toward the patient. At baseline, sociodemographic and clinical data were collected. Clinical data include the Clinical Assessment Interview for Negative Symptoms (CAINS) (27), a clinician-rated 13-item interview on a five-point scale, ranging from zero to four, assessing feelings of pleasure, motivation, and emotional expression. Further, the degree of treatment resistance was assessed with the Maudsley Staging Method for treatment resistant depression (MSM) (28), with a maximum score of 15, assessing duration, symptom severity, and failed treatment attempts of the depressive episode, as well as add-on psychotropic medications and electroconvulsive treatment. Patients' overall psychiatric symptoms were assessed using the clinical-rated Brief Psychiatric Rating Scale (BPRS), a seven-point scale, ranging from one to seven, with 24 items (29). Patients rated their overall health on the EQ-5D VAS, a visual analog scale ranging from 0 to 100 (30), and their depressive symptoms on the Montgomery-Åsberg Depression Rating Scale (MADRS-S) (31), a seven -point scale, ranging from zero to six with nine items. Controls did not receive any iTBS and only underwent baseline assessments. Both the cognitive test battery and the fNIRS acquisition took place at baseline 1 day before treatment start, and once again 4 weeks later. This time interval was chosen to test for potential delayed treatment effects and to synchronize with a neuroimaging study (32).

### Repetitive Transcranial Magnetic Stimulation

The rTMS procedure has been described in detail elsewhere (19, 33). In short, the iTBS treatment was performed using the Cool D-B80 A/P butterfly coil (MagVenture, Denmark), designed for sham-controlled stimulation with one of the two identically looking coil sides being internally shielded to prevent the magnetic field from spreading. Treatment was given twice daily on weekdays with a 15 min break between the treatment sessions (34), aiming at 10 treatment days resulting in 20 iTBS sessions at target intensity. Target intensity was defined as 90% of resting foot motor threshold (35). As rTMS of non-motor areas does not systematically change the motor cortical excitability (36), the determination of the motor threshold was performed only at baseline. Treatment was delivered over the DMPFC following MRI-guided neuronavigation (TMS Navigator, Localite, Bonn, Germany), aiming at the dorsal anterior cingulate cortex (ACC) defined as *x* = 0, *y* = 30, *z* = 30 in the Montreal Neurological Institute coordinates (37) (Supplementary Figure 1). As shown in Hayward et al. (38), the ACC may be reached via rTMS over the medial PFC. Each iTBS session comprised 40 trains of stimulation (2 s on, 8 s off), each train consisting of 10 bursts at 5 Hz and each burst consisting of three biphasic pulses delivered at 50 Hz. For all patients, transcutaneous electrical nerve stimulation (TENS) electrodes were applied medially on the forehead directly beneath the TMS coil. However, only in the sham iTBS allocation, patients received a mild TENS with a maximum current of 4 mA, proportional to stimulation intensity and synchronous with the described rTMS pulses to mimic the sensation of active stimulation. The sham side of the coil was shielded to prevent magnetic stimulation to reach the cortex. No TENS current was applied in the active iTBS allocation.

### Cognitive Tests

The test battery consisted of the Trail Making Test A and B (39), Rey Auditory Verbal Learning Test (RAVLT) (40), Animal Naming Test (41), Digit Symbol Coding Test (42), Sternberg Memory Test (43), Emotional Stroop Test (44), and Corsi Block Tapping Test (45). Except for RAVLT and Animal Naming Test, all tests were administered using the software Inquisit 4 (2015) with computerized adaptations of the tests from the online Millisecond Test Library^1^. Table 1 lists the cognitive tests in the order they were conducted, and details on cognitive domain, duration, outcome, maximum score, and direction of results for each test. All participants undertook the cognitive tests in the same order, at all time points, reflecting increasing difficulty.

**Table 1:** Cognition domain, time or trial number, outcome measure, maximum score and result direction for each cognitive test used in the cognitive test battery.

**Test**	**Domain**	**Time/** **Trials**	**Outcome**	**Maximum score**	**Direction**
Trail Making Test A and B	Attention, processing speed	2 trails	Test completion speed of Trail B (in s)	N/A	Negative
RAVLT	Verbal memory and learning	15 items	Remembered words (sum of parts I-III)	45	Positive
Animal Naming Test	Verbal fluency	60 s	Number of words	N/A	Positive
Digit Symbol Coding Test	Attention, processing speed	120 s	Total score	144	Positive
Sternberg Memory Test	Numerical working memory	21 trials	Proportion of correct trials	100	Positive
Emotional Stroop Test	Inhibitory control	125 trials	RT to negative words minus RT to neutral words (in ms)	N/A	Negative
Corsi Block Tapping Test	Visuospatial working memory	16 trials	Correct trials * blockspan level	144	Positive

*RAVLT, Rey Auditory Verbal Learning Test; RT, Reaction time; N/A, not applicable. A “positive” direction means that higher values indicate higher performance; a “negative” direction means that higher values indicate lower performance*.

#### Trail Making Test A and B

The TMT consists of two parts requiring to connect a set of sequential targets by mouse press as fast as possible, with TMT A consisting of a set of numbers to connect, and TMT B of switching between connecting numbers and letters. The TMT assesses executive functioning, attention and processing speed and was operationalized as TMT B test completion speed in seconds in the present study.

#### Rey Auditory Verbal Learning Test

The RAVLT consists of four parts: in the first three trials, the same set of 15 words is read out by the test instructor and the participant is instructed to immediately verbally recall as many words as possible. The fourth part is a delayed free verbal recall of the word set 30 min later. The RAVLT assesses verbal learning and memory and was operationalized as sum of the first three parts. Two different sets of words were used at baseline and 4 weeks later.

#### Animal Naming Test

The participant is instructed to verbally name as many animals as possible within a 1 min time frame. The test assesses verbal fluency and was operationalized as number of animals named.

#### Digit Symbol Coding Test

The participant is instructed to code via key press as many symbols as possible to their corresponding digit with a digit-symbol pair key within a 2 min time frame. The tests assesses attention and processing speed and was operationalized as number of correctly coded digits.

#### Sternberg Memory Test

Participants are presented with sequences of two to seven digits and subsequently being prompted with a digit, and thus to state by key press whether the digit was in the prior sequence or not. The test assesses numerical working memory and was operationalized as percentage of correct responses.

#### Emotional Stroop Test

Participants are presented with words from five different categories (neutral, negative, positive, aggressive, and color), written in four different colors. Patients are asked to indicate the word color by key press, regardless of the word meaning. The tests assesses inhibitory control and was operationalized as reaction time to negative words subtracted from the reaction time to neutral words (“interference effect”).

#### Corsi Block Tapping Test

Participants are presented with a screen of nine boxes, lighting up sequentially in a predefined order, and ranging in sequence length from two to nine boxes. The participant is instructed to click on the boxes in the correct order. The test assesses visuospatial working memory and was operationalized as the total correct score.

### fNIRS Data Acquisition

The fNIRS signal was obtained from a two-channel NIRS system (NIRO-200 NX, Hamamatsu Photonics, Hamamatsu, Japan). The probe holders were positioned over the left and right forehead, aiming at a site roughly corresponding to the DLPFC, with the detection optode lateral to the emission optode (Supplementary Figure 2). This optode placement allowed for probing effects in the cognitive control network (18), while indirectly even probing for DMPFC-iTBS effects via the targeted corticolimbic network (46). Distance between detection and emission optode was 3.5 cm. Light attenuation changes at three wavelengths, i.e., 735, 810, and 850 nm, were measured using the modified Beer-Lambert law. Based on an estimated differential path length factor of 5.93 according to the NIRS device manual, concentration changes of oxygenated hemoglobin (oxy-Hb), deoxygenated hemoglobin (deoxy-Hb), and total hemoglobin were calculated. In accordance with previous fNIRS studies, oxy-Hb was chosen as main outcome measure, but deoxy-Hb was also reported to complement the hemodynamic picture (47). Sampling frequency was 5 Hz.

Data were collected simultaneously to the cognitive test battery. Signal acquisition started after the patient was seated comfortably in the chair and the fNIRS probe holders were attached. First, an initial 5-min resting-state measurement was conducted with the participant being instructed to sit calmly and rest. Subsequently, the test battery started and was conducted in the above named order, without breaks or resting periods in between cognitive tests. The fNIRS signal acquisition was continuous over the whole test battery duration. Event markers, indicated by button press on the NIRS device by the operating staff, marked the beginning and the end of each cognition test. Due to technical restraints in the experimental software used for the cognition tests, it was not possible to send specific event trigger (i.e., stimulus presentation or data entry by the participant) to the fNIRS device.

### fNIRS Data Analysis

The fNIRS data were analyzed using MATLAB R2020b. For data preprocessing, a low-pass filter with a cutoff frequency of 0.1 Hz was applied to remove potential noise stemming from respiration or heartbeats (48). The fNIRS signal was recorded over a long time period, thus potential changes between tasks being rather slow. To avoid filtering out differences in mean oxy-Hb or deoxy-Hb levels between tasks, no high-pass filter or detrending was applied.

For participant-level analysis, baseline correction was performed by subtracting the mean oxy-Hb or deoxy-Hb signal of the last 60 s within the resting-state measurement, i.e., when the signal had reached equilibrium based on visual inspection of the grouped data, from the filtered fNIRS signal. The fNIRS data were segmented into each cognitive test and contained the initial 60 s of each test performance. With the test length depending on test performance in most tests, we assumed that participants were still somewhat more comparable in their task processing in the beginning rather than in the end of each cognitive test. Given that the Animal Naming Test had a set length of 60 s and thus was the shortest test in the test battery, this length was chosen for all fNIRS data segments. Means were calculated for each cognitive test segment, for both left and right oxy-Hb and deoxy-Hb.

For group-level analysis, the resulting participant-level values were entered into the statistical analysis.

### Statistical Analysis

Statistical analysis was performed using MATLAB R2020b. Demographic, clinical, and cognitive performance characteristics were assessed by means of Mann-Whitney *U*-tests (age, CAINS, EQ-5D VAS, MSM, MADRS-S, BPRS) or χ^2^ tests (sex, education, primary diagnosis, comorbidities, and medication), comparing the two iTBS treatment allocations. To analyze cognitive performance, mean oxy-Hb, and mean deoxy-Hb, linear mixed-effects (LME) models were conducted with the fixed effect factors treatment allocation (active/sham), time (baseline/4 weeks after iTBS), treatment allocation^*^time interaction, and random intercept per subject. To analyze differences between the control group and the patient group (i.e., active and sham iTBS together) at baseline, Mann-Whitney *U*-tests were conducted for cognitive performance and LME models were conducted for mean oxy-Hb, with the fixed effect factors group (controls/patients) and random intercept per subject. Pearson correlation analyses were calculated for (I) patients' anhedonia symptoms and cognitive performance at baseline, (II) patients' negative symptoms and prefrontal oxy-Hb at baseline, (III) patients' cognitive performance and concurrent oxy-Hb at baseline, and (IV) the controls' cognitive performance and concurrent oxy-Hb. The alpha level was set to 0.05 for all statistical tests.

## Results

### Demographic and Clinical Characteristics

After study inclusion, one patient allocated to active iTBS terminated study participation, resulting in 51 patients (23 male, 28 female, mean age = 30) and 55 controls (18 male, 37 female, mean age = 30). Twenty-five patients were randomized to active iTBS, and 26 patients to sham iTBS. Controls had a slightly higher education level but did not differ from patients with respect to age or sex ratio (Table 2). Among patients, there were no baseline differences between the two treatment allocations in either demographic or clinical variables (Table 2).

**Table 2:** Baseline demographic and clinical characteristics [Mean (SD)] of controls and patients, and the patients further divided into sham and active iTBS treatment allocation, and the respective statistical tests.

	**Controls** ** (*n* = 55)**	**Patients** ** (*n* = 51)**	***p***	**Sham** ** (*n* = 26)**	**Active** ** (*n* = 25)**	***p***
Age, years	30.20 (10.55)	29.53 (9.31)	0.874	29.04 (8.73)	30.04 (9.84)	0.821
Sex, *n* male:female	18:37	23:28	0.191	12:14	11:14	0.877
CAINS	–	29.18 (7.63)		30.92 (7.21)	27.36 (7.63)	0.105
EQ-5D VAS	–	34.08 (15.49)		34.38 (14.44)	33.76 (16.50)	0.962
MADRS-S	–	29.81 (7.57)		30.42 (6.96)	29.20 (8.10)	0.604
BPRS subscale	–	20.92 (4.54)		21.19 (4.19)	20.64 (5.04)	0.925
MSM	–	10.06 (1.85)		10.44 (2.00)	9.68 (1.59)	0.181
Education, *n*			0.050			0.127
9th year completed^*^	2	9		6	3	
12th year completed	32	28		16	12	
Higher education	21	14		4	10	
Primary diagnosis, *n*	–					0.382
Depressive episode		28		13	15	
Recurrent depression		18		9	9	
Bipolar depression		5		4	1	
Comorbidities, *n*	–					
Anxiety disorder		21		12	9	0.461
ADHD/ADD		11		8	3	0.173
Medication, *n*	–					
Antidepressants		41		19	22	0.291
Antidopaminergic drugs		10		6	4	0.726
Mood stabilizers		14		8	6	0.588
Stimulants		8		7	1	0.050
No medication		2		0	2	0.235

† *contains data by one patient (in the sham iTBS allocation) who had <9 years of education*.

*CAINS, Clinical Assessment Interview for Negative Symptoms; EQ-5D VAS, self-rated health status; MADRS-S, Montgomery Åsberg Depression Rating Scale, self-rating version; BPRS, Brief Psychiatric rating scale, subscale mood disturbance including the following items: “depression,” “anxiety,” “suicidality,” “guilt,” “suspiciousness,” and “self-neglect;” MSM, Maudsley Staging Method for Treatment Resistant Depression*.

### Baseline Cognitive Performance (Patients vs. Healthy Controls)

At baseline, when comparing the patient group (*n* = 51) with the healthy control group, Mann-Whitney *U*-tests showed a difference in cognitive performance between groups, with lower performance scores for the patients in the Trail Making Test (*p* = 0.026), the RAVLT (*p* < 0.001), the Animal Naming Test (*p* = 0.004), and the Digit Symbol Substitution Test (*p* = 0.016) (Table 3). There were no performance differences between patients and controls in the Sternberg Memory Test (*p* = 0.463), the Emotional Stroop Test (*p* = 0.557), and the Corsi Block Tapping Test (*p* = 0.571).

**Table 3:** Cognitive performance [Mean (SD)] for controls and patients (at baseline), and the patients further divided into sham and active iTBS treatment allocation (at baseline and 4 weeks later), and the respective statistical tests.

	**Baseline**					**4 weeks later**		
	**Controls** ** (*n* = 55)**	**Patients** ** (*n* = 51)**	**Mann-Whitney *U*-tes^†^**	**Sham** ** (*n* = 26)**	**Active** ** (*n* = 25)**	**Sham** ** (*n* = 26)**	**Active** ** (*n* = 25)**	**Linear mixed-effect model^‡^**
Trail Making Test	62.56 (26.52)	73.91 (30.73)	*p = 0.026*	74.89 (30.22)	72.89 (31.22)	65.15 (25.39)	72.79 (54.37)	n.s.
Rey Auditory Verbal Learning Test	30.96 (4.98)	27.47 (6.10)	*p <0.001*	26.92 (5.95)	28.04 (6.19)	27.00 (6.89)	28.04 (5.70)	n.s.
Animal Naming Test	27.78 (6.51)	24.06 (6.12)	*p = 0.004*	23.04 (5.91)	25.12 (6.17)	24.88 (5.96)	26.12 (7.31)	n.s.
Digit Symbol Coding Test	57.24 (15.88)	49.69 (18.22)	*p = 0.016*	50.88 (16.44)	48.44 (19.83)	56.64 (20.22)	56.80 (14.94)	n.s.
Sternberg Memory Test	91.15 (11.39)	92.59 (10.19)	n.s.	91.24 (13.00)	93.99 (5.67)	93.37 (9.87)	95.76 (4.53)	n.s.
Emotional Stroop Test	−4.11 (79.98)	7.15 (113.92)	n.s.	−23.02 (116.80)	38.53 (104.00)	−13.69 (104.56)	−26.03 (143.46)	n.s.
Corsi Block Tapping Test	64.07 (22.61)	61.45 (21.80)	n.s.	60.50 (22.29)	62.44 (21.23)	64.31 (22.77)	64.96 (28.51)	n.s.

*For detailed test information see Table 1*.

† *Mann-Whitney U-Test for baseline comparison of controls (n = 55) and patients (n = 51)*,

‡ Linear Mixed-Effect (LME) models with the fixed effect factors treatment allocation (active/sham) and time (baseline/4 weeks later iTBS). Reported are the p-values for each group*time interaction.

### Cognitive Performance Following DMPFC-iTBS (Active vs. Sham iTBS)

Cognitive performance for each cognitive test at baseline and 4 weeks later is shown in Table 3. LME models for each cognition test showed no treatment allocation^*^time interaction, reflecting no differences in trajectories between active and sham iTBS (Table 3).

### Baseline Prefrontal Blood Oxygenation (Patients vs. Healthy Controls)

**Figure 1:**
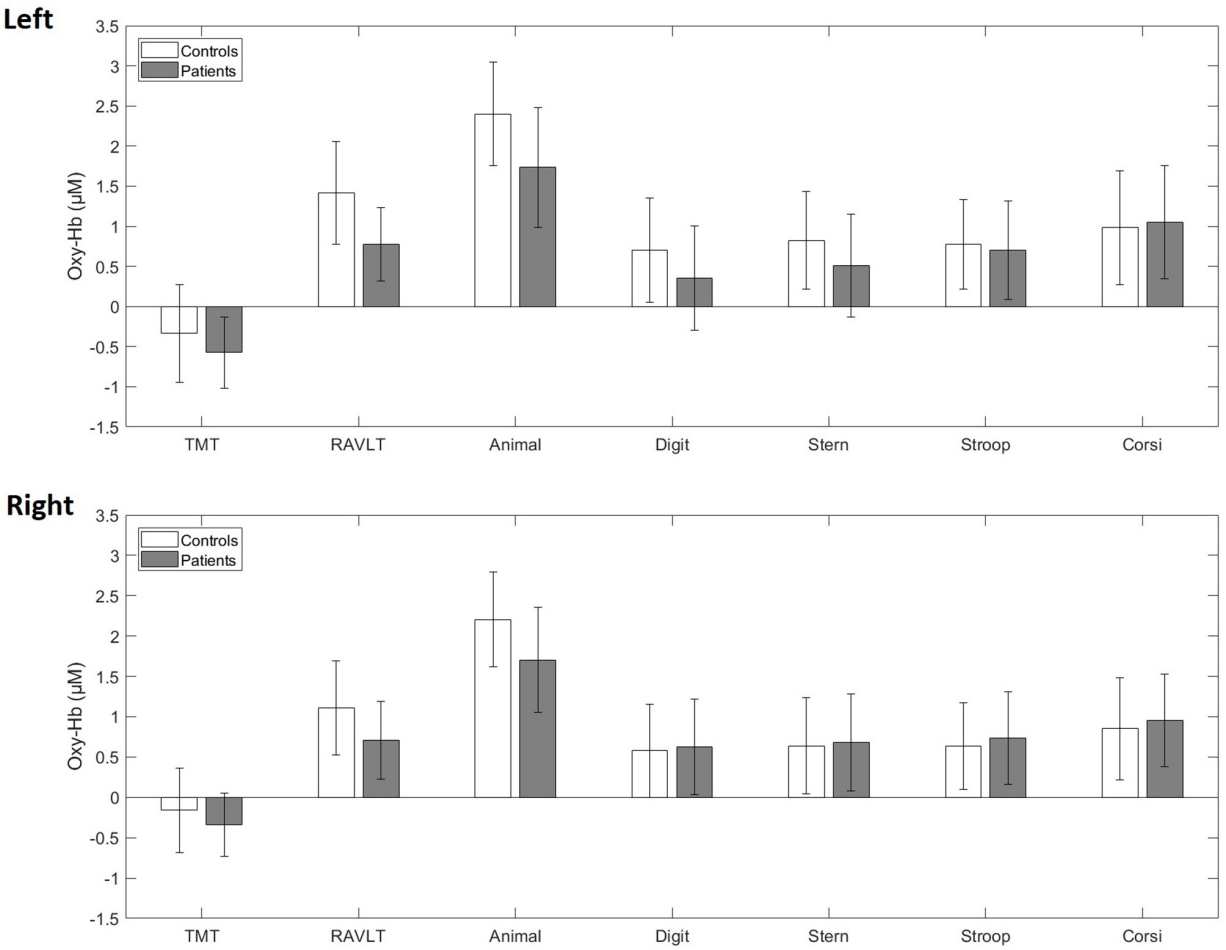
Averaged blood oxygenation (oxy-Hb) and confidence intervals in patients (gray) and controls (white) during the cognitive test battery at baseline in the left and right fNIRS channel. TMT, Trail-Making-Test; RAVLT, Rey Auditory Verbal Learning Test; Animal, Animal Naming Test; Digit, Digit Symbol Coding Test; Stern, Sternberg Memory Test; Stroop, Emotional Stroop Test; Corsi, Corsi Block Tapping Test.

At baseline, when comparing the whole patient group (*n* = 51) with the healthy control group (*n* = 55), LME models for each cognitive test showed no main effect of group, reflecting no mean oxy-Hb change differences between patients and controls (Figure 1, Supplementary Figures 3, 4). Similarly, LME models showed no mean deoxy-Hb differences between the groups (Supplementary Figure 5).

### Prefrontal Blood Oxygenation Following DMPFC-iTBS (Active vs. Sham iTBS)

**Figure 2:**
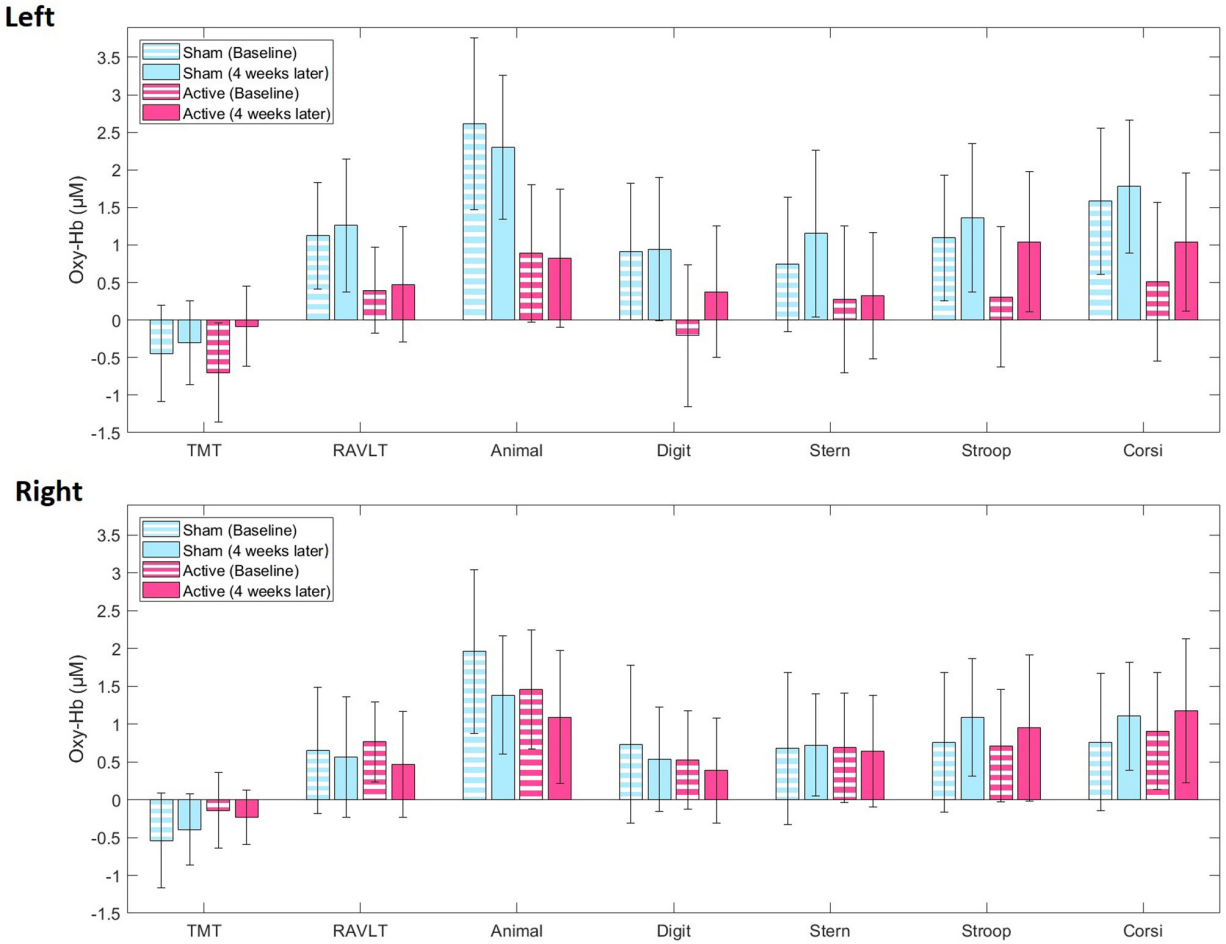
Averaged blood oxygenation (oxy-Hb) and confidence intervals in the active (pink) and sham (blue) iTBS group during the cognitive test battery at baseline (stripes) and 4 weeks later (full color) in the left and right fNIRS channel. TMT, Trail-Making-Test; RAVLT, Rey Auditory Verbal Learning Test; Animal, Animal Naming Test; Digit, Digit Symbol Coding Test; Stern, Sternberg Memory Test; Stroop, Emotional Stroop Test; Corsi, Corsi Block Tapping Test.

For concurrent mean oxy-Hb levels, LME models showed no main effect of treatment allocation or time, and no treatment allocation^*^time interaction for either cognitive test (Supplementary Table 1), reflecting no differences in trajectories from baseline to 4 weeks later between active and sham iTBS (Figure 2, Supplementary Figures 6, 7). Similarly, LME models showed no mean deoxy-Hb differences over the treatment course between active and sham iTBS (Supplementary Figure 8).

### Correlations

Pearson correlation analyses between baseline patients' negative symptoms and cognitive performance showed negative correlations for the RAVLT (*r* = −0.29, *p* = 0.037) and the Animal Naming Test (*r* = −0.28, *p* = 0.049), and trend-level correlations for the Trail-Making-Test (*r* = 0.27, *p* = 0.055), reflecting worse cognitive performance with more negative symptoms (Supplementary Table 2). However, no correlation was found between negative symptoms and cognitive performance assessed 4 weeks later (data not shown). For baseline prefrontal oxy-Hb during cognitive performance, there was a negative correlation with patients' negative symptoms at oxy-Hb during the Trail-Making-Test (*r* = −0.29, *p* = 0.042), reflecting lower cortical activation with more negative symptoms (Supplementary Table 3). However, such correlation was no longer observed after the iTBS treatment (data not shown). No correlations were found between baseline cognitive performance and concurrent oxy-Hb for either patients (Supplementary Table 4) or healthy controls (Supplementary Table 5).

## Discussion

To the best of our knowledge, this is the first sham-controlled study to investigate how iTBS over the DMPFC affects cognitive performance, and concurrent prefrontal blood oxygenation changes. While patients at baseline did show impaired cognitive performance in several cognition tests, we did not see the predicted concurrent prefrontal hypoactivity reflected in the fNIRS signal. Further, we did not detect any differences between active and sham iTBS regarding cognitive performance or blood oxygenation changes over time. Negative symptoms correlated with cognitive performance to some extent, however there were no correlations between cognitive performance and blood oxygenation.

Our cognition results showed that active iTBS over DMPFC did not affect cognitive performance. The Trail Making Test performance has been reported to be positively affected by left DLPFC-rTMS (11) as well as DMPFC-iTBS (14). Improvement has also been observed in the Wisconsin Card sorting after iTBS to DLPFC. However, our results are in line with several studies showing no difference in cognitive performance following high frequency rTMS over left DLPFC, for example in the Digit Symbol Substitution Test (49), the Stroop Color Test (50), the Stroop Color-Word Interference Test (51), and the digit span (52). Further, a recent meta-analysis of the mostly small sample size studies, revealed no overall DLPFC-rTMS effects on attention, executive functioning, processing speed, verbal fluency, verbal learning, and social cognition were reported (53). DMPFC and DLPFC are engaged in the same cognitive control network (18), and it is possible that this network is less sensitive to manipulation by rTMS/iTBS than hitherto suggested.

Our fNIRS results showed that active iTBS over the DMPFC did not modulate blood oxygenation during cognitive testing. As the DLPFC is suggested as the main hub in executive functioning (54), we chose this region for recording of the blood oxygenation signal. With the DMPFC also being part in the cognitive control network (18), this allowed us to probe for indirect cognitive network effects. In a fNIRS study with iTBS over left DLPFC in panic disorder patients, active iTBS did not modulate prefrontal activity during a verbal fluency task (24). This is in line with our observations during the Animal Naming Test in the present study.

We did not observe a prefrontal hypoactivity when comparing the fNIRS signal of our depressed patients to the controls. This is somewhat surprising, as previous fMRI studies showed hypoactivity of prefrontal regions during various cognitive tasks, including a verbal fluency task (55) and a working memory task (56). Also with fNIRS, such hypofrontality has been reported in depression (57), and even with a similar two-channel device as the one used in our study (58). However, descriptively, our patients did exhibit lower prefrontal blood oxygenation throughout the cognitive testing. This trend not reaching significance however might partly be due to that in our study, we measured fNIRS continuously over a relatively long timespan, without applying an event-related paradigm as the majority of studies investigating working memory (57). Similarly, this might at least partly explain the lack of association between the fNIRS signal and cognitive performance.

Though patients did not show a cognitive improvement following the active compared to sham iTBS treatment course, it is important to stress that neither did active iTBS over the DMPFC cause worsened cognitive performance, making it likely to be a cognitively safe treatment option in this patient sample. rTMS targeting the DLPFC has not been reported to have cognitive side-effects such as temporary memory loss (59) which is the case for electroconvulsive therapy (5), and our study extends this observation to be also valid for iTBS over DMPFC.

This study is subject to some limitations. First, the observation that accelerated protocols yield a more rapid treatment response has been questioned by a recent study (60) and it is possible that the targeted treatment duration of 10 days thus was too short to yield a substantial treatment effect on cognitive functioning. In this context, the 15 min intersession interval used in our study might have been too short to increase cortical excitability (61). While longer intervals of 60 min are more reliably linked to excitatory effects (62), there are also reports of 15 min intervals yielding increased excitability (34). As of now, the optimal intersession interval length between two daily sessions to yield maximum excitatory effects is yet to be determined. Second, we cannot fully exclude that the sham iTBS might have been partly active, as has been suggested by a recent rTMS study with the same coil (63). Third, the fNIRS signal may be sensitive also to signal changes from the skin blood flow, thus picking up psychophysiological arousal due to the cognitive performance situation (64). Fourth, as the patients had psychotropic medication this may have affected the NIRS-signal (65), making comparisons with healthy controls less reliable. However, as the patients' medications were unchanged 1 month before and during the blinded randomized phase of the trial, it is unlikely that the medication introduced any systematic bias in the comparison between sham and active iTBS. Finally, it should be noted that our two-channel fNIRS device only obtained signals from a limited brain region.

This sham-controlled study did not detect any changes in the prefrontal blood oxygenation during cognitive performance, and no changes in cognitive performance following a full treatment course of active iTBS over the DMPFC in depression. Together with our previous findings (19), showing a treatment effect for negative symptoms, such as anhedonia, while not causing more side effects, such as self-reported memory deficits or fatigue, than sham iTBS, we conclude that the treatment is likely to be considered cognitively safe in this sample of patients with depression.

## Data Availability Statement

The raw data supporting the conclusions of this article will be made available by the authors, without undue reservation.

## Ethics Statement

The studies involving human participants were reviewed and approved by Central Ethical Review Board, Uppsala University. The patients/participants provided their written informed consent to participate in this study.

## Author Contributions

JP, MG, WW, CW, and RB: conception and design of the study. WS, JP, MG, and RB: acquisition and analysis of data. WS, JP, MG, WW, CW, and RB: drafting the manuscript or figures. All authors contributed to the article and approved the submitted version.

## Conflict of Interest

The authors declare that the research was conducted in the absence of any commercial or financial relationships that could be construed as a potential conflict of interest.

## Footnotes

^1^https://www.millisecond.com/download/library/

## Supplementary Material

Supplementary Figure 1 Coil placement over the dorsomedial prefrontal cortex (DMPFC). For detailed coil information see Methods.

Supplementary Figure 2 Position of the fNIRS probe holders, aiming at a site roughly corresponding to the dorsolateral prefrontal cortex (DLPFC), with the detection optode lateral to the emission optode.

Supplementary Figure 3 Averaged blood oxygenation (oxy-Hb) and confidence intervals in patients (blue) and controls (yellow) during the cognitive test battery at baseline in the left fNIRS channel. Displayed are the initial 60 s of each cognitive test. Gaps between tests stem from time spent for test instruction. TMT, Trail-Making-Test; RAVLT, Rey Auditory Verbal Learning Test; Animal, Animal Naming Test; Digit, Digit Symbol Coding Test; Sternberg, Sternberg Memory Test; Stroop, Emotional Stroop Test; Corsi, Corsi Block Tapping Test.

Supplementary Figure 4 Averaged blood oxygenation (oxy-Hb) and confidence intervals in patients (blue) and controls (yellow) during the cognitive test battery at baseline in the right fNIRS channel. Displayed are the initial 60 s of each cognitive test. Gaps between tests stem from time spent for test instruction. TMT, Trail-Making-Test; RAVLT, Rey Auditory Verbal Learning Test; Animal, Animal Naming Test; Digit, Digit Symbol Coding Test; Sternberg, Sternberg Memory Test; Stroop, Emotional Stroop Test; Corsi, Corsi Block Tapping Test.

Supplementary Figure 5 Averaged blood oxygenation (deoxy-Hb) and confidence intervals in patients (gray) and controls (white) during the cognitive test battery at baseline in the left and right fNIRS channel. TMT, Trail-Making-Test; RAVLT, Rey Auditory Verbal Learning Test; Animal, Animal Naming Test; Digit, Digit Symbol Coding Test; Stern, Sternberg Memory Test; Stroop, Emotional Stroop Test; Corsi, Corsi Block Tapping Test.

Supplementary Figure 6 Averaged blood oxygenation (oxy-Hb) and confidence intervals in the active (red) and sham (blue) iTBS group during the cognitive test battery at baseline (lighter color) and 4 weeks later (darker color) in the left fNIRS channel. Displayed are the initial 60 s of each cognitive test. Gaps between tests stem from time spent for test instruction. TMT, Trail-Making-Test; RAVLT, Rey Auditory Verbal Learning Test; Animal, Animal Naming Test; Digit, Digit Symbol Coding Test; Sternberg, Sternberg Memory Test; Stroop, Emotional Stroop Test; Corsi, Corsi Block Tapping Test.

Supplementary Figure 7 Averaged blood oxygenation (oxy-Hb) and confidence intervals in the active (red) and sham (blue) iTBS group during the cognitive test battery at baseline (lighter color) and 4 weeks later (darker color) in the right fNIRS channel. Displayed are the initial 60 s of each cognitive test. Gaps between tests stem from time spent for test instruction. TMT, Trail-Making-Test; RAVLT, Rey Auditory Verbal Learning Test; Animal, Animal Naming Test; Digit, Digit Symbol Coding Test; Sternberg, Sternberg Memory Test; Stroop, Emotional Stroop Test; Corsi, Corsi Block Tapping Test.

Supplementary Figure 8 Averaged blood oxygenation (deoxy-Hb) and confidence intervals in the active (pink) and sham (blue) iTBS group during the cognitive test battery and baseline (stripes) and 4 weeks later (full color) in the left and right fNIRS channel. TMT, Trail-Making-Test; RAVLT, Rey Auditory Verbal Learning Test; Animal, Animal Naming Test; Digit, Digit Symbol Coding Test; Stern, Sternberg Memory Test; Stroop, Emotional Stroop Test; Corsi, Corsi Block Tapping Test.

Supplementary Table 1 Depicted are the results of linear mixed-effects models for concurrent oxy-Hb assessed during a cognitive test battery, i.e., the T- and *p*-values of the treatment allocation (active, sham) x time (baseline, 4 weeks later) interactions. RAVLT, Rey Auditory Verbal Learning Test. fNIRS, functional near-infrared spectroscopy.

Supplementary Table 2 Baseline correlations between patients' negative symptoms as assessed by the Clinical Assessment Interview for Negative Symptoms (CAINS) and cognitive performance at baseline. RAVLT, Rey Auditory Verbal Learning Test. Significant correlations are marked with an asterisk.

Supplementary Table 3 Baseline correlation between patients' negative symptoms as assessed by the Clinical Assessment Interview for Negative Symptoms (CAINS) and prefrontal oxy-Hb during cognitive performance at baseline. RAVLT, Rey Auditory Verbal Learning Test. Significant correlations are marked with an asterisk.

Supplementary Table 4 Baseline correlation between patients' cognitive performance and concurrent prefrontal oxy-Hb at baseline. RAVLT, Rey Auditory Verbal Learning Test.

Supplementary Table 5 Correlations between controls' cognitive performance and concurrent prefrontal oxy-Hb. RAVLT, Rey Auditory Verbal Learning Test.
