# Supplementary figures and images for: Unchanged Cognitive Performance and Concurrent Prefrontal Blood Oxygenation After Accelerated Intermittent Theta-Burst Stimulation in Depression: A Sham-Controlled Study
Source: Front Psychiatry. 2021 Jun 30;12:659571. doi: 10.3389/fpsyt.2021.659571 (PMC8278060; doi:10.3389/fpsyt.2021.659571)

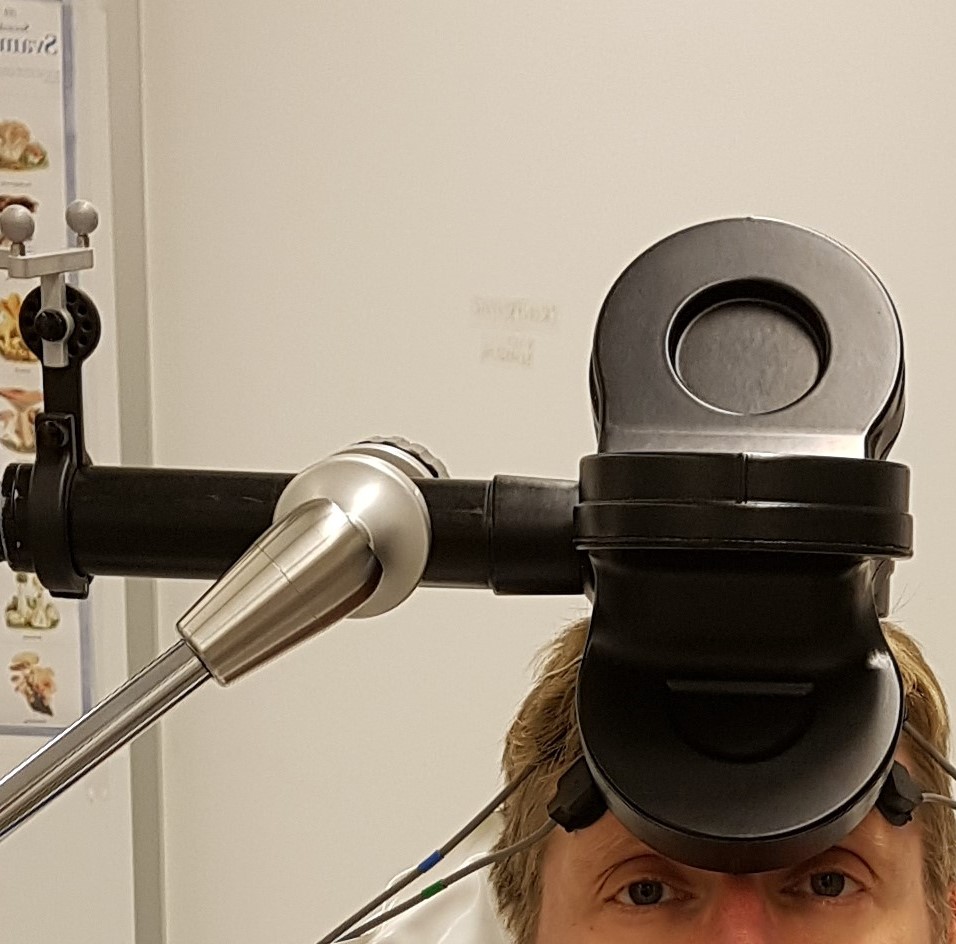

Supplement: Supplementary Figure 1 — Coil placement over the dorsomedial prefrontal cortex (DMPFC). For detailed coil information see Methods. [file Image_1.jpg]

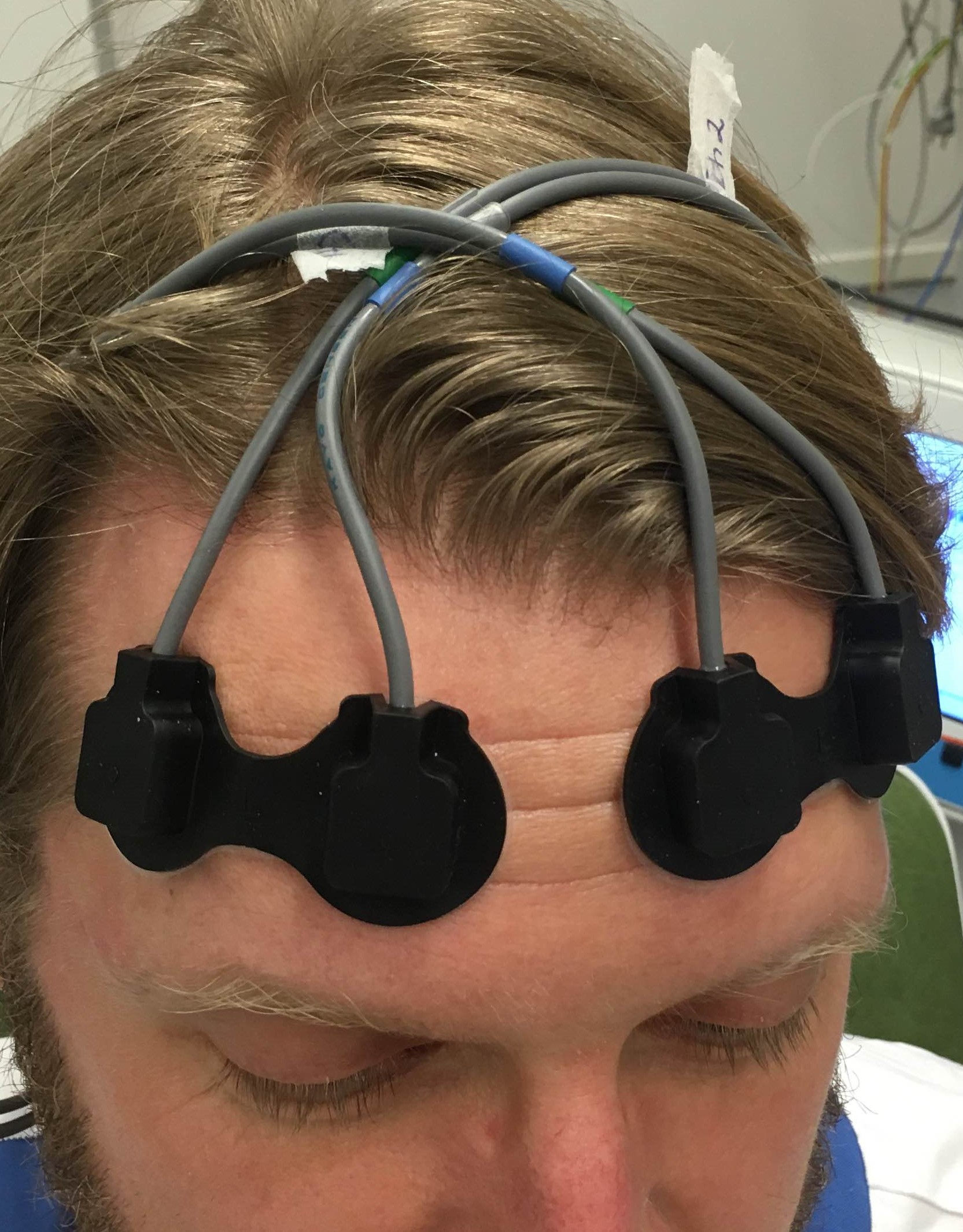

Supplement: Supplementary Figure 2 — Position of the fNIRS probe holders, aiming at a site roughly corresponding to the dorsolateral prefrontal cortex (DLPFC), with the detection optode lateral to the emission optode. [file Image_2.jpg]

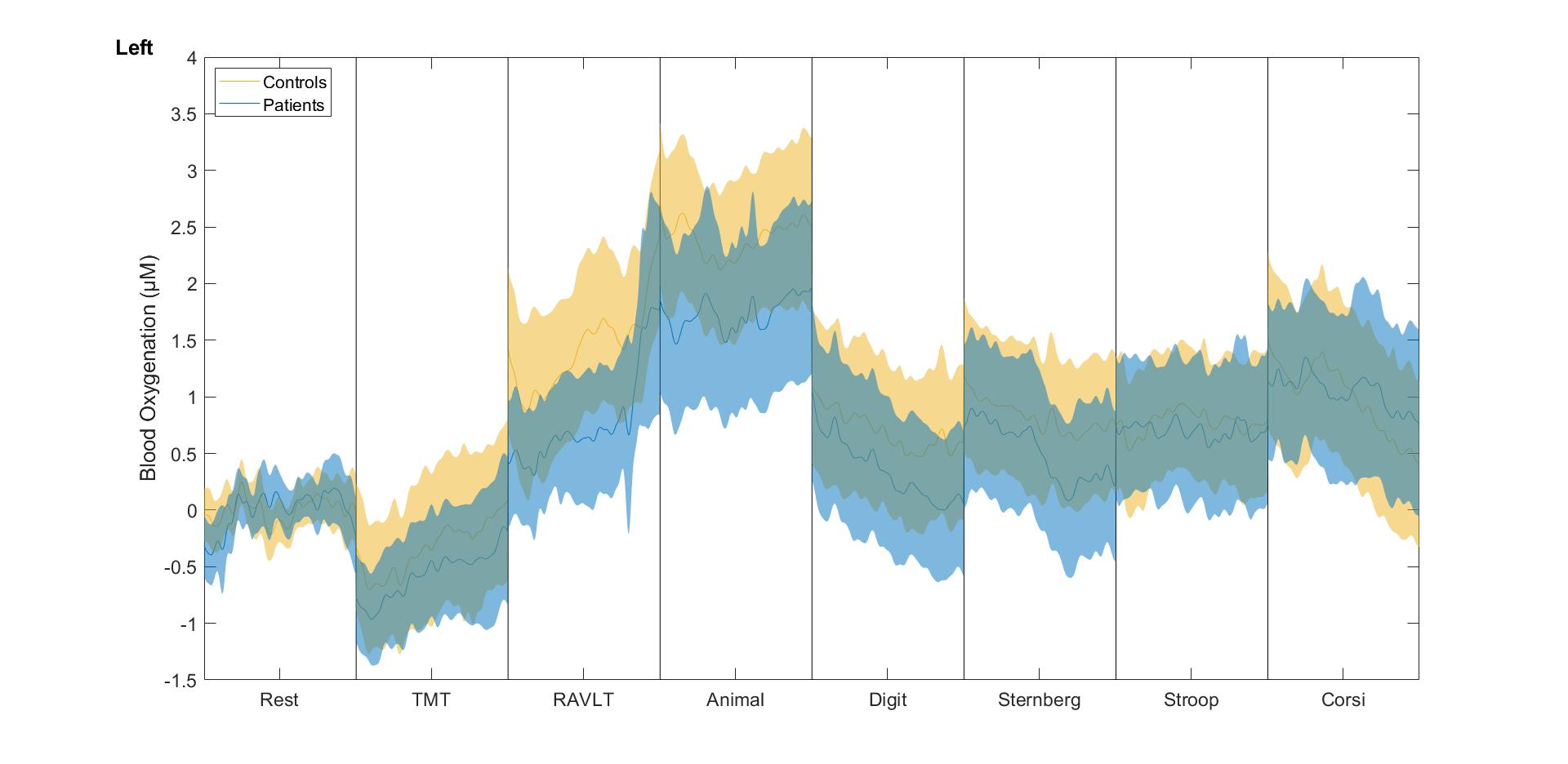

Supplement: Supplementary Figure 3 — Averaged blood oxygenation (oxy-Hb) and confidence intervals in patients (blue) and controls (yellow) during the cognitive test battery at baseline in the left fNIRS channel. Displayed are the initial 60 s of each cognitive test. Gaps between tests stem from time spent for test instruction. TMT, Trail-Making-Test; RAVLT, Rey Auditory Verbal Learning Test; Animal, Animal Naming Test; Digit, Digit Symbol Coding Test; Sternberg, Sternberg Memory Test; Stroop, Emotional Stroop Test; Corsi, Corsi Block Tapping Test. [file Image_3.jpg]

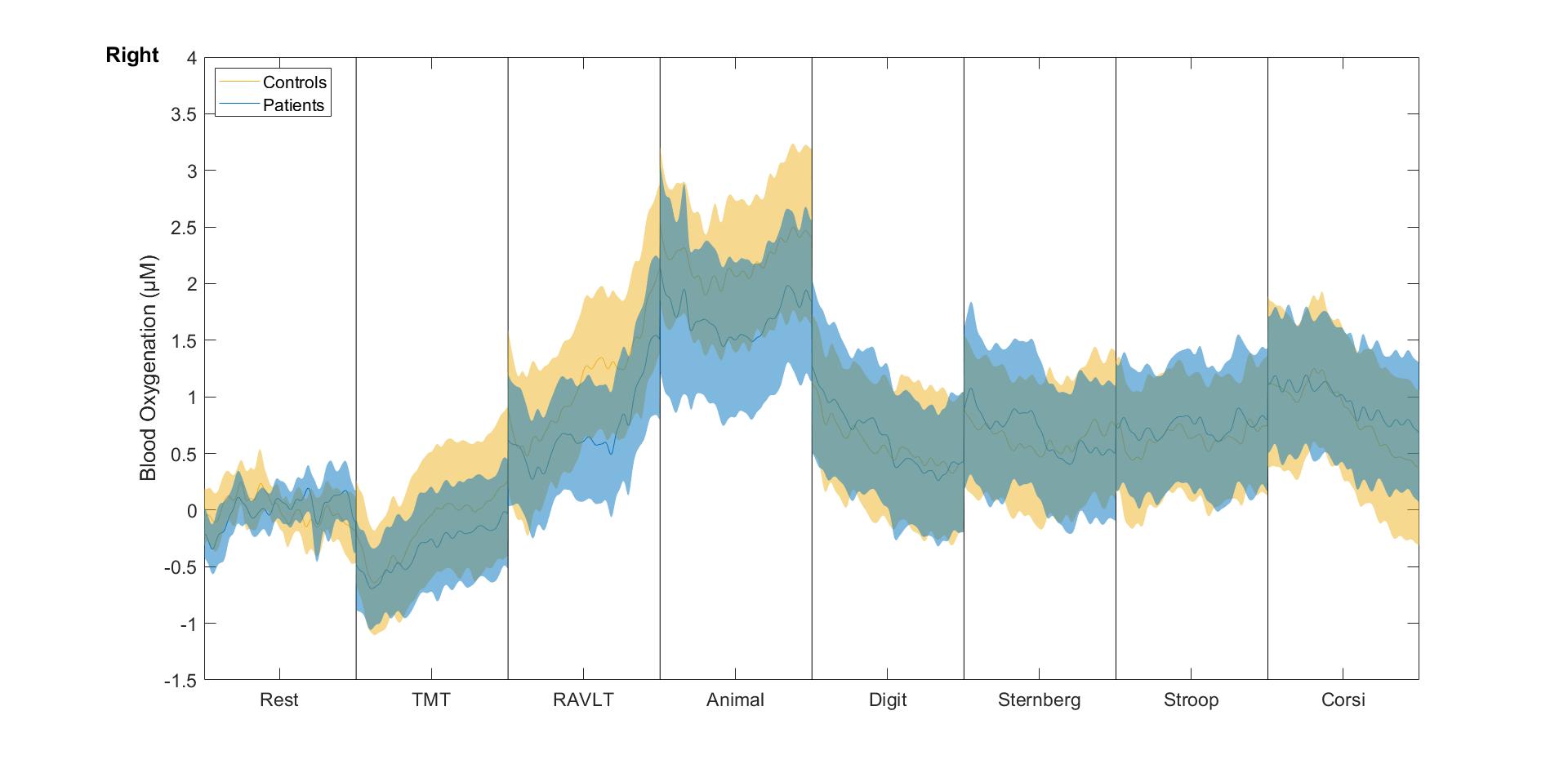

Supplement: Supplementary Figure 4 — Averaged blood oxygenation (oxy-Hb) and confidence intervals in patients (blue) and controls (yellow) during the cognitive test battery at baseline in the right fNIRS channel. Displayed are the initial 60 s of each cognitive test. Gaps between tests stem from time spent for test instruction. TMT, Trail-Making-Test; RAVLT, Rey Auditory Verbal Learning Test; Animal, Animal Naming Test; Digit, Digit Symbol Coding Test; Sternberg, Sternberg Memory Test; Stroop, Emotional Stroop Test; Corsi, Corsi Block Tapping Test. [file Image_4.jpg]

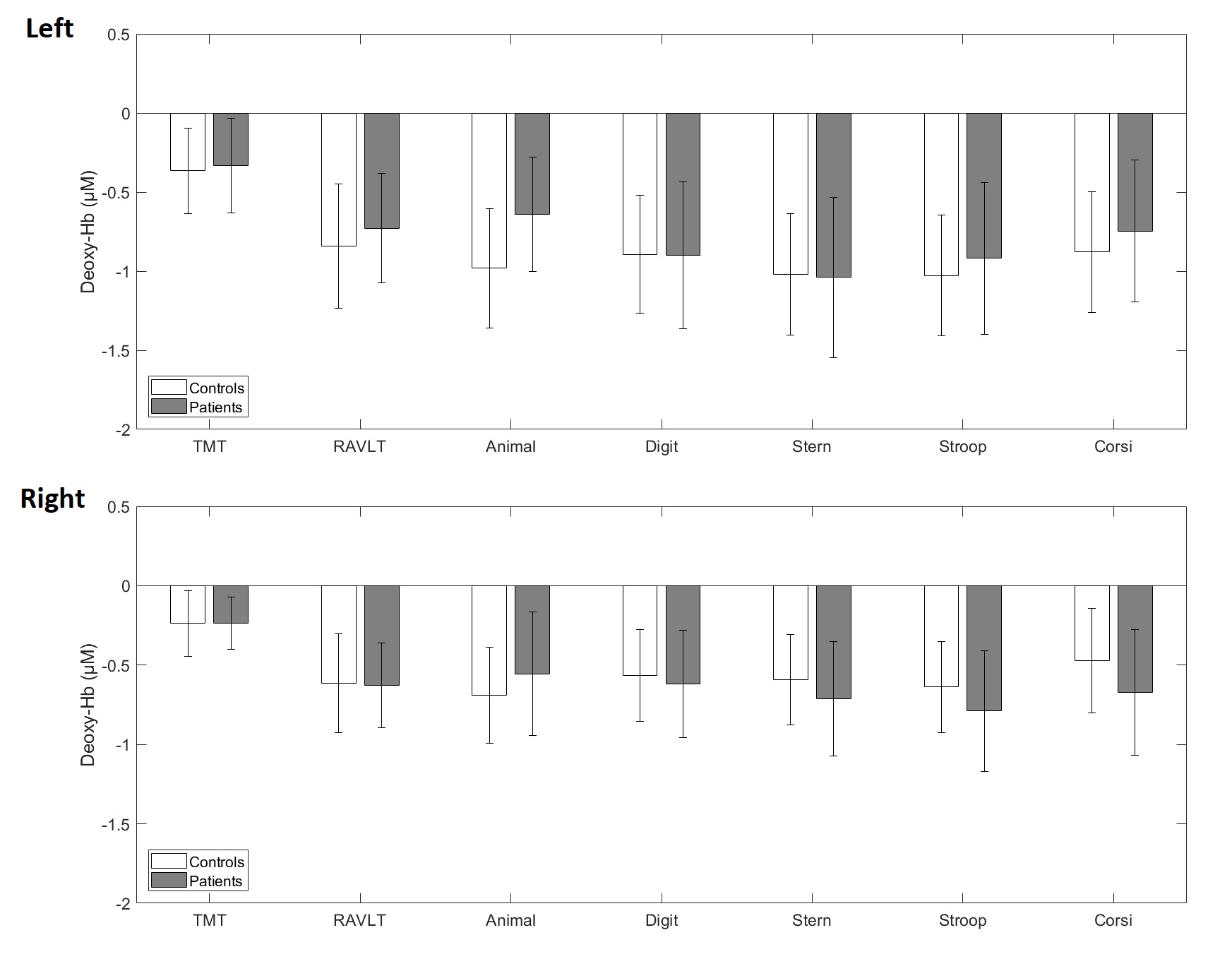

Supplement: Supplementary Figure 5 — Averaged blood oxygenation (deoxy-Hb) and confidence intervals in patients (gray) and controls (white) during the cognitive test battery at baseline in the left and right fNIRS channel. TMT, Trail-Making-Test; RAVLT, Rey Auditory Verbal Learning Test; Animal, Animal Naming Test; Digit, Digit Symbol Coding Test; Stern, Sternberg Memory Test; Stroop, Emotional Stroop Test; Corsi, Corsi Block Tapping Test. [file Image_5.jpg]

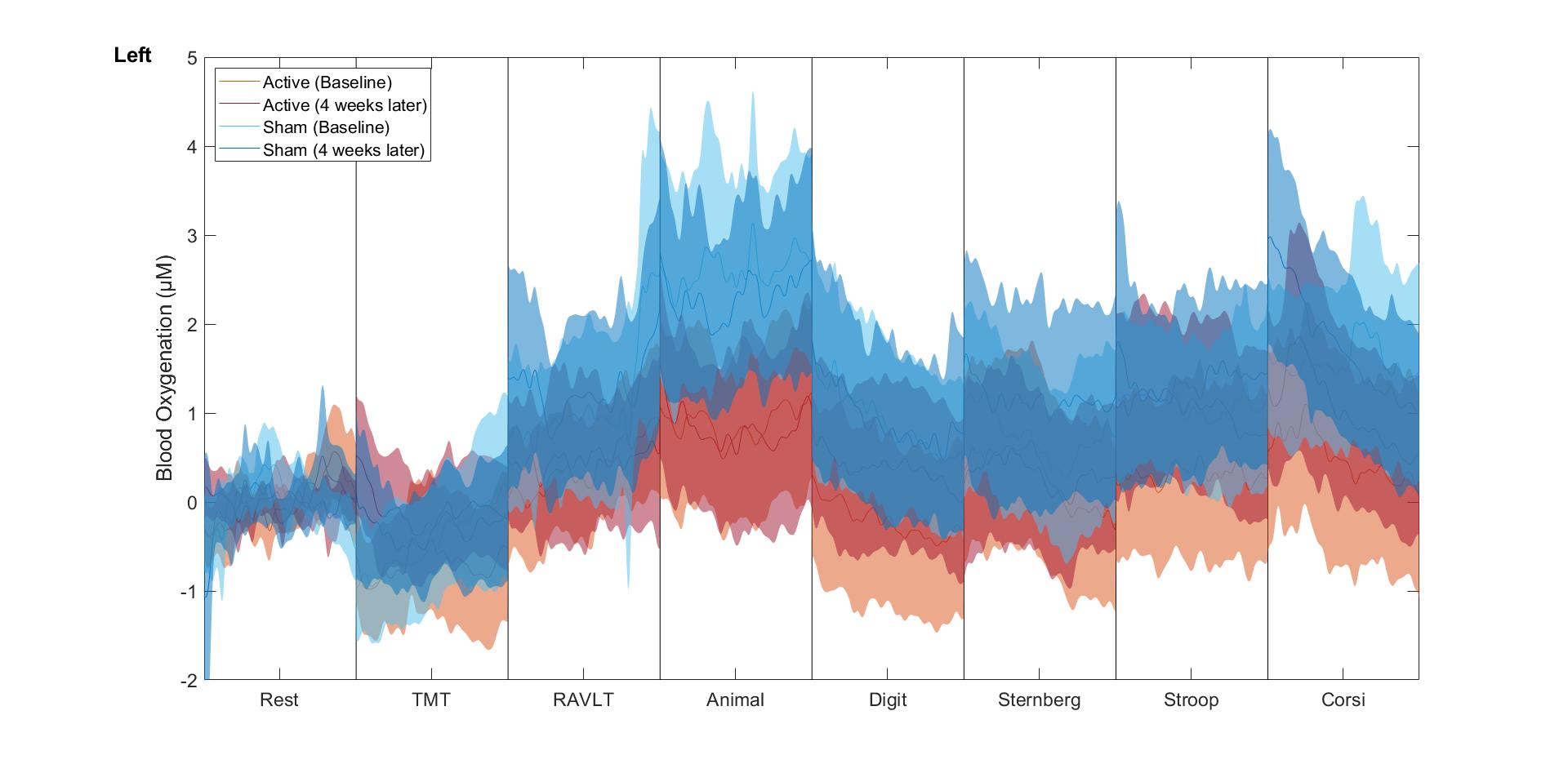

Supplement: Supplementary Figure 6 — Averaged blood oxygenation (oxy-Hb) and confidence intervals in the active (red) and sham (blue) iTBS group during the cognitive test battery at baseline (lighter color) and 4 weeks later (darker color) in the left fNIRS channel. Displayed are the initial 60 s of each cognitive test. Gaps between tests stem from time spent for test instruction. TMT, Trail-Making-Test; RAVLT, Rey Auditory Verbal Learning Test; Animal, Animal Naming Test; Digit, Digit Symbol Coding Test; Sternberg, Sternberg Memory Test; Stroop, Emotional Stroop Test; Corsi, Corsi Block Tapping Test. [file Image_6.jpg]

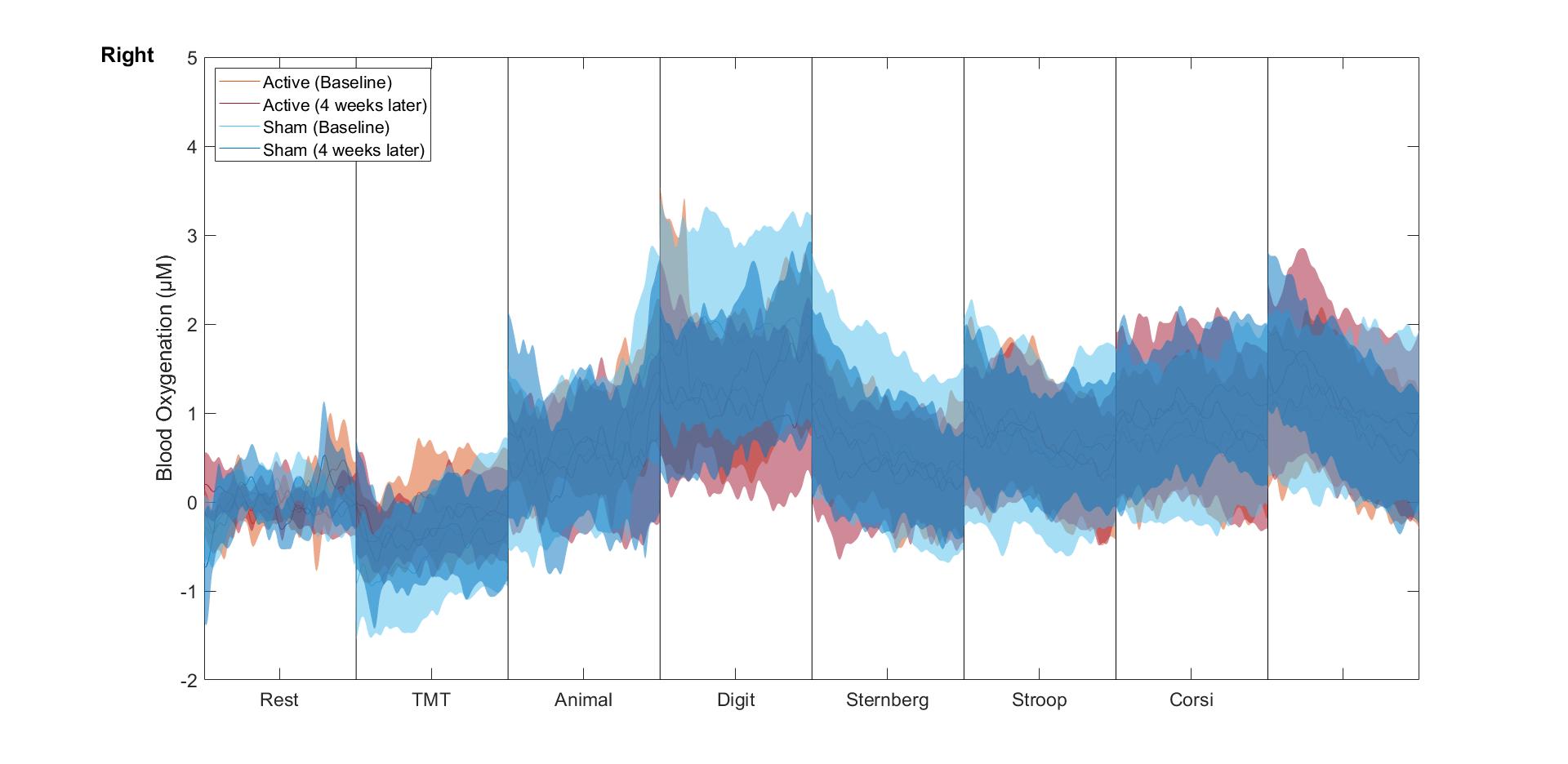

Supplement: Supplementary Figure 7 — Averaged blood oxygenation (oxy-Hb) and confidence intervals in the active (red) and sham (blue) iTBS group during the cognitive test battery at baseline (lighter color) and 4 weeks later (darker color) in the right fNIRS channel. Displayed are the initial 60 s of each cognitive test. Gaps between tests stem from time spent for test instruction. TMT, Trail-Making-Test; RAVLT, Rey Auditory Verbal Learning Test; Animal, Animal Naming Test; Digit, Digit Symbol Coding Test; Sternberg, Sternberg Memory Test; Stroop, Emotional Stroop Test; Corsi, Corsi Block Tapping Test. [file Image_7.jpg]

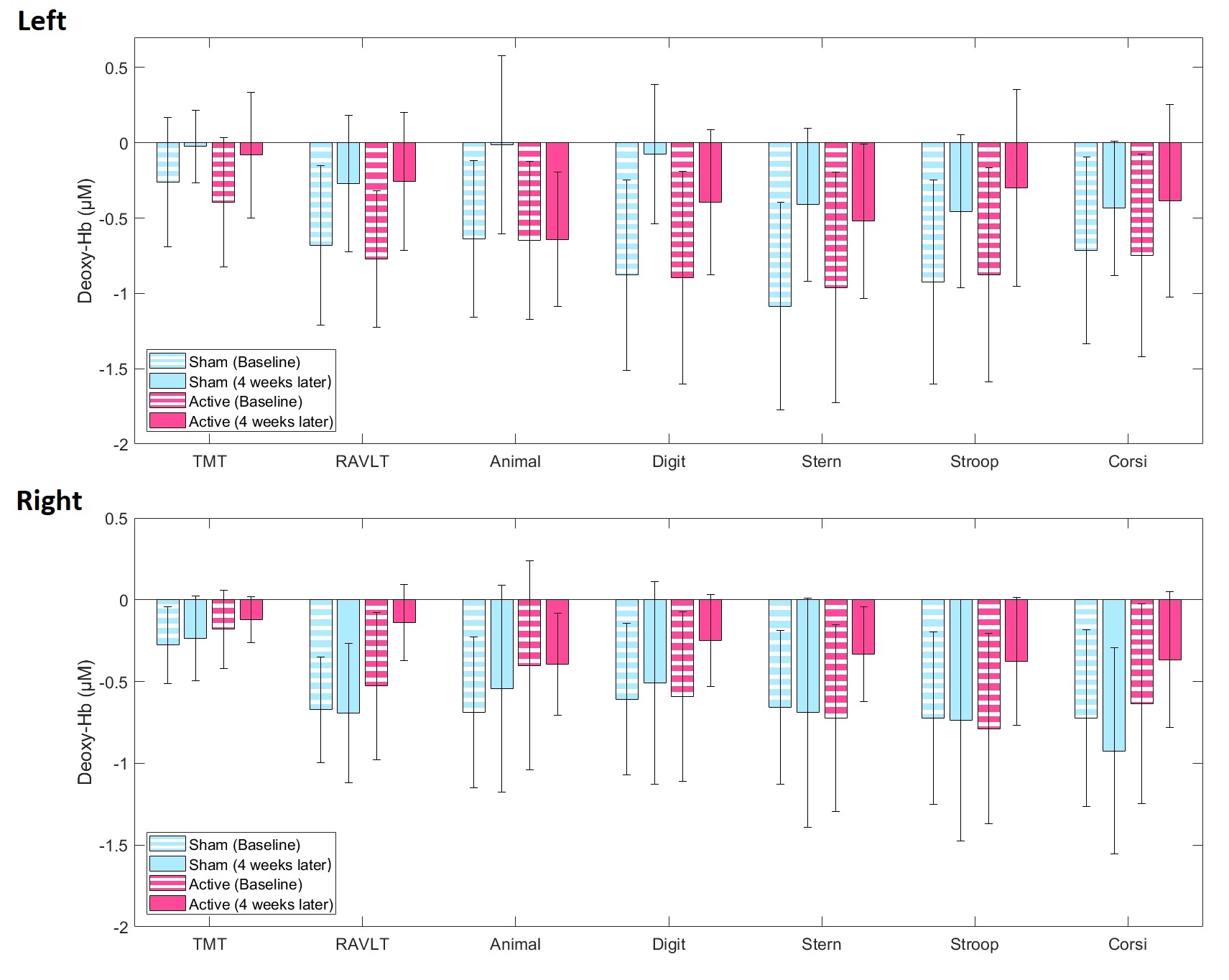

Supplement: Supplementary Figure 8 — Averaged blood oxygenation (deoxy-Hb) and confidence intervals in the active (pink) and sham (blue) iTBS group during the cognitive test battery and baseline (stripes) and 4 weeks later (full color) in the left and right fNIRS channel. TMT, Trail-Making-Test; RAVLT, Rey Auditory Verbal Learning Test; Animal, Animal Naming Test; Digit, Digit Symbol Coding Test; Stern, Sternberg Memory Test; Stroop, Emotional Stroop Test; Corsi, Corsi Block Tapping Test. [file Image_8.jpg]
