# Supplementary material for: Unchanged Cognitive Performance and Concurrent Prefrontal Blood Oxygenation After Accelerated Intermittent Theta-Burst Stimulation in Depression: A Sham-Controlled Study
Source: Front Psychiatry. 2021 Jun 30;12:659571. doi: 10.3389/fpsyt.2021.659571 (PMC8278060; doi:10.3389/fpsyt.2021.659571)
Supplement: Supplementary Table 1 — Depicted are the results of linear mixed-effects models for concurrent oxy-Hb assessed during a cognitive test battery, i.e., the T- and p-values of the treatment allocation (active, sham) x time (baseline, 4 weeks later) interactions. RAVLT, Rey Auditory Verbal Learning Test. fNIRS, functional near-infrared spectroscopy. [file Table_1.docx]

*Supplementary Table 1.* Depicted are the results of linear mixed-effects models for concurrent oxy-Hb assessed during a cognitive test battery, i.e. the T- and p-values of the treatment allocation (active, sham) x time (baseline, 4 weeks later) interactions.

| **Cognition tests** | **Blood oxygenation** | | | |
| --- | --- | --- | --- | --- |
|  | Left fNIRS channel | | Right fNIRS channel | |
|  | T | *p* | T | *p* |
| Trail Making Test | 1.29 | .202 | -.66 | .514 |
| RAVLT | -.10 | .925 | -.35 | .726 |
| Animal Naming Test | .54 | .592 | .48 | .635 |
| Digit Symbol Coding Test | .88 | .379 | .09 | .927 |
| Sternberg Memory Test | -.34 | .732 | -.12 | .903 |
| Emotional Stroop Test | .77 | .447 | -.08 | .934 |
| Corsi Block Tapping Test | .48 | .630 | .21 | .836 |

*RAVLT:* Rey Auditory Verbal Learning Test. *fNIRS*: functional near-infrared spectroscopy
