# Supplementary material for: Unchanged Cognitive Performance and Concurrent Prefrontal Blood Oxygenation After Accelerated Intermittent Theta-Burst Stimulation in Depression: A Sham-Controlled Study
Source: Front Psychiatry. 2021 Jun 30;12:659571. doi: 10.3389/fpsyt.2021.659571 (PMC8278060; doi:10.3389/fpsyt.2021.659571)
Supplement: Supplementary Table 2 — Baseline correlations between patients' negative symptoms as assessed by the Clinical Assessment Interview for Negative Symptoms (CAINS) and cognitive performance at baseline. RAVLT, Rey Auditory Verbal Learning Test. Significant correlations are marked with an asterisk. [file Table_2.docx]

*Supplementary Table 2.* Baseline correlations between patients’ negative symptoms as assessed by the Clinical Assessment Interview for Negative Symptoms (CAINS) and cognitive performance at baseline.

| **Cognition tests** | **CAINS-score** |  |
| --- | --- | --- |
|  | r | *p* |
| Trail Making Test | .27 | .055 |
| RAVLT | -.29 | .037 * |
| Animal Naming Test | -.28 | .049 * |
| Digit Symbol Coding Test | -.13 | .348 |
| Sternberg Memory Test | -.24 | .096 |
| Emotional Stroop Test | .22 | .117 |
| Corsi Block Tapping Test | -.11 | .426 |

*RAVLT:* Rey Auditory Verbal Learning Test. Significant correlations are marked with an asterisk.
