# Supplementary material for: Unchanged Cognitive Performance and Concurrent Prefrontal Blood Oxygenation After Accelerated Intermittent Theta-Burst Stimulation in Depression: A Sham-Controlled Study
Source: Front Psychiatry. 2021 Jun 30;12:659571. doi: 10.3389/fpsyt.2021.659571 (PMC8278060; doi:10.3389/fpsyt.2021.659571)
Supplement: Supplementary Table 3 — Baseline correlation between patients' negative symptoms as assessed by the Clinical Assessment Interview for Negative Symptoms (CAINS) and prefrontal oxy-Hb during cognitive performance at baseline. RAVLT, Rey Auditory Verbal Learning Test. Significant correlations are marked with an asterisk. [file Table_3.docx]

*Supplementary Table 3.* Baseline correlation between patients’ negative symptoms as assessed by the Clinical Assessment Interview for Negative Symptoms (CAINS) and prefrontal oxy-Hb during cognitive performance at baseline.

|  | **CAINS-score** | | | |
| --- | --- | --- | --- | --- |
|  | Left oxy-Hb | | Right oxy-Hb | |
|  | r | *p* | r | *p* |
| Trail Making Test | -.29 | .042* | -.17 | .226 |
| RAVLT | -.12 | .416 | -.03 | .840 |
| Animal Naming Test | -.21 | .164 | .09 | .538 |
| Digit Symbol Coding Test | -.20 | .184 | .12 | .425 |
| Sternberg Memory Test | -.20 | .157 | .04 | .780 |
| Emotional Stroop Test | -.16 | .280 | -.00 | .978 |
| Corsi Block Tapping Test | -.12 | .400 | .04 | .769 |

*RAVLT:* Rey Auditory Verbal Learning Test. Significant correlations are marked with an asterisk.
