# Supplementary material for: Unchanged Cognitive Performance and Concurrent Prefrontal Blood Oxygenation After Accelerated Intermittent Theta-Burst Stimulation in Depression: A Sham-Controlled Study
Source: Front Psychiatry. 2021 Jun 30;12:659571. doi: 10.3389/fpsyt.2021.659571 (PMC8278060; doi:10.3389/fpsyt.2021.659571)
Supplement: Supplementary Table 4 — Baseline correlation between patients' cognitive performance and concurrent prefrontal oxy-Hb at baseline. RAVLT, Rey Auditory Verbal Learning Test. [file Table_4.docx]

*Supplementary Table 4.* Baseline correlation between patients’ cognitive performance and concurrent prefrontal oxy-Hb at baseline.

| **Cognition tests** | **Left oxy-Hb** | | **Right oxy-Hb** | |
| --- | --- | --- | --- | --- |
|  | r | *p* | r | *p* |
| Trail Making Test | .06 | .678 | .07 | .641 |
| RAVLT | -.16 | .285 | -.19 | .199 |
| Animal Naming Test | -.12 | .437 | -.13 | .380 |
| Digit Symbol Coding Test | -.13 | .391 | -.17 | .254 |
| Sternberg Memory Test | .21 | .136 | .06 | .704 |
| Emotional Stroop Test | .01 | .970 | .15 | .310 |
| Corsi Block Tapping Test | .12 | .423 | .12 | .390 |

*RAVLT:* Rey Auditory Verbal Learning Test.
