# Supplementary material for: Unchanged Cognitive Performance and Concurrent Prefrontal Blood Oxygenation After Accelerated Intermittent Theta-Burst Stimulation in Depression: A Sham-Controlled Study
Source: Front Psychiatry. 2021 Jun 30;12:659571. doi: 10.3389/fpsyt.2021.659571 (PMC8278060; doi:10.3389/fpsyt.2021.659571)
Supplement: Supplementary Table 5 — Correlations between controls' cognitive performance and concurrent prefrontal oxy-Hb. RAVLT, Rey Auditory Verbal Learning Test. [file Table_5.docx]

*Supplementary Table 5.* Correlations between controls’ cognitive performance and concurrent prefrontal oxy-Hb.

| **Cognition tests** | **Left oxy-Hb** | | **Right oxy-Hb** | |
| --- | --- | --- | --- | --- |
|  | r | *p* | r | *p* |
| Trail-Making-Test | .13 | .366 | .11 | .412 |
| RAVLT | -.21 | .134 | -.11 | .423 |
| Animal Naming Test | -.09 | .506 | -.15 | .278 |
| Digit Symbol Coding Test | -.17 | .214 | -.03 | .860 |
| Sternberg Memory Test | -.20 | .148 | -.11 | .439 |
| Emotional Stroop Test | -.21 | .136 | -.05 | .725 |
| Corsi Block Tapping Test | .04 | .781 | .13 | .363 |

*RAVLT:* Rey Auditory Verbal Learning Test.
